# Immigrant women’s experience of labor and birth

**DOI:** 10.1590/1980-220X-REEUSP-2022-0444en

**Published:** 2024-01-05

**Authors:** Lilian Salem Supimpa, Silvana Regina Rossi Kissula de Souza, Naiane Ribeiro Prandini, Dayane Andreatta, Tatiane Herreira Trigueiro, Bibiana Amaral Paviani

**Affiliations:** 1 Universidade Federal do Paraná, Departamento de Enfermagem, Curitiba, PR, Brazil.

**Keywords:** Culture, Emigrants and Immigrants, Obstetric Nursing, Parturition, Midwifery, Cultura, Emigrantes e inmigrantes, Enfermería Obstétrica, Parto, Partería, Cultura, Emigrantes e Imigrantes, Enfermagem Obstétrica, Parto, Tocologia

## Abstract

**Objective:**

To describe the childbirth experience of immigrant women in maternity hospitals in southern Brazil.

**Method:**

Descriptive, qualitative study, Hybrid Thematic Oral History method, in two public maternity hospitals in Curitiba-PR; semi-structured interviews collected from March to December 2020. Analysis followed the proposed method.

**Results:**

The seven interviewees - collaborators immigrated from Venezuela, Haiti and Tunisia. Relevant themes emerged: 1) Surprises and feelings during the childbirth process, pointing out preferences, unexpected birth outcomes, factors related to the higher incidence of C-section and descriptions of sensations and feelings; 2) The care perceived by women and memories of experiences in the country of origin, with reports of previous childbirth experience, difficulties in the current childbirth and perceptions of the care received.

**Conclusion:**

The childbirth process was experienced with expectation, accessing feelings and memories. The positive childbirth experience was favored by team care, participation in decision-making, well-informed prenatal care, bonding with the care team, effective communication and evidence-based obstetric practices. Challenges were perceived regarding cultural sensitivity in care.

## RESUMO

**Objetivo:**

Descrever a experiência de parto de mulheres imigrantes em maternidades do sul do Brasil.

**Método:**

Estudo descritivo, qualitativo, método História Oral Temática Híbrida, em duas maternidades públicas de Curitiba-PR; entrevistas semiestruturadas coletadas de março a dezembro de 2020. Análise seguiu o método proposto.

**Resultados:**

As sete colaboradoras imigraram da Venezuela, Haiti e Tunísia. Emergiram como temas relevantes: 1) As surpresas e sentimentos durante o processo de parto, apontando preferências, desfechos de parto inesperados, fatores relacionados à maior incidência de cesariana e descrição de sensações e sentimentos; 2) O cuidado percebido pelas mulheres e as memórias de experiências no país de origem, com relatos de experiência prévia de parto, dificuldades no parto atual e percepções do cuidado recebido.

**Conclusão:**

O processo de parto foi experienciado com expectativa, acessando sentimentos e memórias. A experiência positiva de parto foi favorecida pelo cuidado da equipe, participação na tomada de decisão, assistência pré-natal bem-informada, vínculo com equipe assistencial, comunicação efetiva e práticas obstétricas baseadas em evidência. Foram percebidos desafios sobre sensibilidade cultural na assistência.

## RESUMEN

**Objetivo:**

Describir la experiencia del parto de las mujeres inmigrantes en las maternidades del sur de Brasil.

**Método:**

Estudio descriptivo, cualitativo, utilizando el método de Historia Oral Temática Híbrida, en dos maternidades públicas de Curitiba-PR; entrevistas semiestructuradas recogidas de marzo a diciembre de 2020. El análisis siguió el método propuesto.

**Resultados:**

Los siete colaboradores inmigraron de Venezuela, Haití y Túnez. Surgieron como temas relevantes: 1) Sorpresas y sentimientos durante el proceso de parto, señalando preferencias, resultados inesperados del parto, factores relacionados con la mayor incidencia de cesárea y descripción de sensaciones y sentimientos; 2) La atención percibida por las mujeres y recuerdos de experiencias en el país de origen, con experiencia de parto anterior, dificultades en el parto actual y percepción de los cuidados recibidos.

**Conclusión:**

El proceso del parto se vivió con expectación, accediendo a sensaciones y recuerdos. Una experiencia positiva del parto se vio favorecida por la atención en equipo, la participación en la toma de decisiones, una atención prenatal bien informada, el establecimiento de vínculos con el equipo asistencial, la comunicación eficaz y las prácticas obstétricas basadas en pruebas. Se percibieron retos relacionados con la sensibilidad cultural en la atención.

## INTRODUCTION

Pregnancy and birth are events laden with customs and values in many cultures, bringing complex changes for women. The individual experience is the result of the interaction of the collective meanings provided by their culture^(1)^, which means that women from different contexts bring with them knowledge about their bodies, their babies and how to care for both, and this knowledge intersects with scientific knowledge^(2)^. In this scenario, reproductive health care for immigrant women should receive attention. This group of migrant people is subject to vulnerability, precarious living conditions and different forms of violence produced by inequality, their social, gender, sexual and reproductive role, in the periods leading up to transit, during transit and in the process of adapting to the new country^(3)^.

There are events that shape or even favor obstetric violence against immigrant women during pregnancy, childbirth and puerperium^(2,4)^, and lack of knowledge of the culture and communication are just one of the main challenges faced^(4)^. Thus, for effective and comprehensive care to be provided, communication strategies are essential, considering that health services are also an environment for intercultural exchanges, since the values, beliefs and practices related to the pregnancy-puerperal cycle are present in care from the perspective of both women, migrants or not, and health professionals^(4)^.

In the Brazilian context, between 2011 and 2020, there was an increase in the number of migrant women, who represent 363,321 of the total 986,919 migrations in the country in the period. The characteristic of registered women is that they are young and of working age, with the highest number of female registrations in that decade, 127,027 women, aged between 25 and 40. This migratory movement means that the health system is mainly looking for care related to pregnancy, childbirth and the puerperium^(5)^.

However, despite the pertinency and urgency of this topic, there are gaps in the production of studies relating the experience of pregnancy, childbirth and birth among immigrant women, with knowledge of cultural competencies among the health team that assists them^(6)^. Based on the scenario of female immigration in Brazil and aiming to promote the humanization of labor and birth care, this study sought to contribute to improving care for immigrant women during labor and birth care, and aimed to describe the experience of immigrant women in the labor and birth process in two public maternity hospitals in southern Brazil.

## METHOD

### Study Design

This is a descriptive, qualitative research using the Thematic Oral History method. Oral History allows people to access their memories and the meaning they give to life events through the narratives of the people interviewed; it seeks to give culturally minority groups a space to validate their life experiences and relate cultural values, conceiving social meaning and a common identity, so that the bond between one person and another happens through the connection arising from the experiences^(7)^. Thematic Oral History is conceived as one in which the interviews have a pre-established theme in the project to be researched with the aim of elucidating conflicting and unknown situations related to it. It is based on the idea that if we research a given social, demographic or ethnic group, this group will reveal a consensus on a given topic^(7)^.

### Study Site

The research was done in Curitiba, capital of the state of Paraná, in the southern region of Brazil, a state that concentrated 23,695 of the registrations of immigrant women in the decade 2011–2020 and is one of the main entry points for this population into the country^(5)^. The research site encompassed two maternity hospitals belonging to the Hospital de Clínicas Complex of the Federal University of Paraná (UFPR CHC in the Portuguese acronym), which provided care to pregnant women stratified as usual risk, intermediate risk and high risk in the city and neighboring municipalities, according to state agreements.

The Victor Ferreira do Amaral Maternity Hospital, with its own physical structure, provided care for normal-risk pregnant women, as well as other low-risk obstetric and gynecological procedures and family planning activities. The second maternity ward is attached to the physical structure of the UFPR CHC, and the maternity ward is a specific wing, with its own entrance, serving high-risk pregnant women. The structure includes obstetric emergency care, genetic counseling, high-risk Ob-Gyn and prenatal care, an obstetric surgical center, rooming-in, a Neonatal Intensive Care Unit (NICU) and a gynecology ward, with a multi-professional clinical team, including residency and undergraduate students.

### Population

To select the participants, referred to in Oral History as collaborators, we followed the methodological definitions of community of destination, colony and network. A community of destination means a large number of people who have experienced a critical, dramatic situation in common, so that this experience brings people together, giving rise to community memories^(7)^; in the case of this research, it was “Immigrant women living in the city of Curitiba”. The colony is a cross-section of the community of destination, maintaining the common characteristics of the collaborators; it can be done according to gender, age group, origin, or other similar aspects^(7)^; here it was “Immigrant women who live in the city of Curitiba and have experienced labor and childbirth n the city”. And the network, an even more specific delineation of the colony, made up of people who have experienced a common dramatic situation^(7)^, here “Women who have migrated to Curitiba, who have had the experience of labor and birth in the city, in their diversity of country of origin, religion, family context, route of delivery, among others”.

### Inclusion and Exclusion Criteria

The inclusion criteria were: immigrant women who gave birth in the aforementioned maternity hospitals in 2020; who had their babies between 38 and 42 weeks of gestation, via normal delivery or cesarean section, who were with their newborn in a rooming-in unit or in the NICU, and who could communicate in the following languages: Portuguese, Spanish, English, French and Creole. Exclusion criteria included: participants under the age of 18, without a companion of legal age; who had experienced childbirth due to fetal death; or premature births; participants whose chosen language did not have a translator available in the research team. One participant, whose spoken Creole language had an accent unknown to the researcher-translator, was excluded from the study.

### Data Collection

Data collection took place from March to December 2020, during the COVID-19 pandemic, applying semi-structured interviews, with the guiding question being “Tell me about your experience of giving birth in this maternity hospital”. The interviews were recorded, generating a total of 66 minutes and 81 seconds of audio, which were transcribed into 19 pages of text and were collected by the researcher, the main author of this article, who at the time was a master’s student and obstetric nurse, having, in her experience, been an immigrant pregnant woman and parturient in another country. In addition to the main researcher, a Haitian undergraduate nursing student at UFPR and a postgraduate student who is a member of the research group took part in the transcription. Information about the entire project was recorded in the field notebook^(7)^.

To recruit the collaborators, the services were contacted to locate women who met the criteria; then there was the pre-interview, the first contact with the collaborator, in the rooming-in ward during puerperal hospitalization, before discharge. Once she had been invited and agreed to take part in the research, identification and contact details were collected and the time and place for the interview were scheduled. The interviews took place as scheduled with each collaborator, conducted by the main author and beginning after a new presentation about the research and the procedures relating to the Informed Consent Form (ICF); they were carried out in a private room in the maternity wards or another hospital environment that guaranteed privacy. The collaborators were invited to remain in contact for feedback after the final version of the text.

### Data Analysis and Processing

After the interview, the data went through the processes proposed by the method: Transcription, when the narrated content is transcribed in its entirety; Textualization, where questions and external noises are excluded and grammatical errors are corrected, the vital tone of the interview is listed, and the phrase spoken by the collaborator is what points to the essence of her discourse; Trans-creation, in which the text is organized so that the main idea is understood without losing the original intention, with the ideas forming a story, with the vital tone as a guide^(7)^.

In Oral History, isolated interviews do not speak for themselves, they are just random texts; a process of analysis is needed to align the accounts and promote an interconnection between them^(7)^. Thus, a thorough reading of each interview was carried out, with a broad view of what the set of narratives revealed about the labor and birth process of immigrant women. After further reading, the relevant themes of the study were extracted: “Surprises and feelings during the childbirth process”, which deals with preferences, unexpected birth outcomes, factors related to the higher incidence of caesarean section, and the description of sensations and feelings; and “The care perceived by women and memories of experiences in the country of origin”, which reveals previous childbirth experiences, difficulties faced in the current childbirth and perceptions of the care received by the multi-professional team during the childbirth process. The analysis was carried out with the relevant themes, because finding these internal “connecting points” allows the researcher to visualize the social dimension of the surrounding context^(7)^.

### Ethical Aspects

Data collection began after approval No. 3.793.262 in 2019 by the Ethics Committee of the Research Department of the CHC UFPR, the Victor Ferreira do Amaral Maternity Hospital and the Department of Obstetrics and Gynecology of the UFPR. The collaborators signed an informed consent form before being interviewed. To preserve anonymity in the narratives, the original name of each participant was replaced by the name of a city in their country of origin, chosen by the participant. The ethical and legal principles of Resolution 466/2012 of the National Health Council^(8)^ were followed. Each collaborator was given the opportunity to access the final text and approve the final version of the Letter of Assignment, either digitally or in person at a location of her choice. The Letter of Assignment is a document inherent to Oral History research and signing it grants approval of the texts as part of the study^(7)^.

## RESULTS

Building of knowledge through the stories of a group of individuals starts from the point of recognizing what binds them, understanding the scenario of where they come from and where they are, and connecting these many points to their narrative and to the narratives of the collective^(7)^.

Seven immigrant women from Venezuela, Haiti and Tunisia took part, speaking Portuguese, Spanish, English, French and Creole. Their period of residence in Brazil ranged from three months to over four years. They were aged between 30 and 38, six were married, and four had completed high school. Regarding parity, two were primiparous and five were multiparous and the main birth was surgical (Table 1). There were no refusals or withdrawals from data collection, but access to hospitalized puerperal women was difficult due to the Covid-19 pandemic safety protocols.

**Table 1:** Characterization of the Collaborators – Curitiba, PR, Brazil, 2020.

Collaborator and date of interview	Caracas 13/03/2020	Carrefour 14/03/2020	Port au Prince 14/03/2020	Tunis 15/03/2020	Maracaibo 18/11/2020	Valencia 23/11/2020	Delmas 01/12/2020
***Vital tone***	Everyone treated me well and was worried about what was happening to me and the baby	I have nothing bad to say about them, because what I didn’t expect, they managed to do. Anyway, it’s all good	I suffered, suffered, suffered, until the other day. I suffered	I was sad, I was scared by the doctor’s words about the risks of a third C-section	I was prepared for something else and when they told me, I said I didn’t want a C-section	I said I didn’t want to give birth, I wanted a caesarean section. I couldn’t bear to go through that	So we have to obey, so that everything goes well for us and our daughter. We believe that the baby has to be born
***Country of origin***	Venezuela	Haiti	Haiti	Tunisia	Venezuela	Venezuela	Haiti
***Time in Brazil***	<1 year	<4 years	<4 years	>1 year	<1 year	>1 year	<4 years
***Age***	30 years	38 years	34 years	38 years	31 years	30 years	30 years
***Marital status***	Married	Married	Married	Married	Single	Married	Married
Parity*	G1	G2P1C1	G1	G7C3A4	G3P1C1A1	G2C2	G2P1C1
***Delivery route***	Normal	C-section	C-section	C-section	C-section	C-section	C-section

Key: *GPCA= G number of pregnancies; P number of deliveries; C C-sections; A abortions.

After trans-creation and analysis of the narratives, two relevant themes emerged: 1) Surprises and feelings during the birth process, showing preferences and unexpected birth outcomes, factors related to the higher incidence of C-sections and the manifestation of sensations and feelings; 2) The care perceived by women and memories of experiences in the country of origin, addressing previous experiences, difficulties in the current birth and perceptions of care received by the multi-professional team.

### Surprises and Feelings During the Birth Process

The birth process is a surprising event and the encounter with the unexpected permeated the narratives. In this respect, three collaborators were surprised by the early delivery after a routine consultation, six by the outcome of the delivery route, others by the lack of control and the unknown that childbirth represented, while for multiparous women this happened during the birth process:

*I arrived here (hospital) just to stabilize my pressure. When I got here, I never imagined that I would have a baby. I always wanted to have the baby by normal delivery. It was a surprise. I wasn’t prepared to have her now. But thanks to the doctors here, she came (Maracaibo).*

With regard to expectations about the route of delivery, two multiparous women, each with a previous caesarean section, chose C-section again as their route of delivery; four others had caesarean sections for emergency indications, the reasons for which were failure of labor progression with altered fetal cardio-tocopheresis, acute fetal distress, maternal hypertension and pre-eclampsia:

*The baby’s head was almost out. But he saw that the baby had pooped inside my belly. So she called another doctor, who said it wasn’t possible to deliver the baby normally. And (pause) I would have to go quickly for the cesarean section (Port-au-Prince).*

It was noticeable that, with the exception of the two multiparous women with previous caesarean sections, the other participants preferred the vaginal delivery route, the only clear reason being the possibility of having the newborn close by in the immediate postpartum period. The reasons given for preferring the surgical route were to prepare help in the family environment and with the children who would be staying at home:

*(...) I didn’t want a normal delivery; I wanted a C- section. To complicate matters, my mother, who was working, had already requested a leave permit (...) I didn’t want to wait the 41 weeks because I had family members who had planned to help me (Valencia).*

The labor and birth process were permeated with emotion. The internal female process, physiological sensations and thoughts evoked feelings and memories that were strongly present during the birth process. Pain was a unanimous theme, regardless of whether the birth was natural or induced, and was also presented as puerperal pain and post-section pain:

*I couldn’t sleep that night. The pain was horrible (...) But I couldn’t stand the pain, I couldn’t stand the pain. The contractions came and the pain came (...) I saw God (laughs), because the pain was unbearable and I wasn’t dilating. They had already done the touch test twice and a brown liquid was coming out. They said I was 2 and there was no more dilation. I didn’t want to endure another touch; it was too painful (Valencia).*

Feelings of fear, sadness, loneliness and unhappiness were reported:

*When they told me they would have to do a C-section because the baby might be in fetal distress. I didn’t believe them. I even cried, because it wasn’t what I wanted. (...) I was scared, because I didn’t want surgery (Maracaibo).*

Many collaborators described the experience positively. The feeling of happiness was especially related to the discharge from hospital and the birth:

*Today I’m feeling happy, I’m happy (...) Now I’m happy. It was a good birth, very good (Port-au-Prince).*

One of the collaborators specifically reported fear related to distrust of the care team made up of students, when she said I was afraid when I saw so many young people (Tunis); another collaborator felt welcomed and cared for when she found herself alone and nauseous during surgery:

*At that moment my husband was away. I was very nervous, even though they were talking to me. When I was feeling sick, they said “it’s normal, it’s normal”. They took a towel because I couldn’t touch anything. I didn’t feel anything, I just felt the urge to vomit. But in my mind, I didn’t want to throw up. I didn’t want to be embarrassed either. The doctor said “you have to stay calm, don’t worry”, and put the towel near my mouth. He changed the towel, rubbed my hand, helped me, took care of everything. If it hadn’t been for them, I think I’d be worse off (Delmas)*

### Women’s Perceived Care and Memories of Experiences in their Country of Origin

The experiences were permeated by memories and comparisons with previous births and obstetric care in the country of origin. The multiparous women who gave birth in other countries unanimously preferred giving birth in Brazil, relating this choice to the free public service and the possibility of enjoying it without a specific visa or health insurance:

*My other birth was normal, in a hospital in Haiti. But I thought it was better here, because you don’t have to pay anything. Everything is free, and it’s very comfortable (Carrefour).*

Negative experiences of previous births were closely linked to expectations of the current birth. They reported poor and derogatory obstetric care in their country of origin, leading them to feel insecure about Brazilian care:

*I waited a few days to seek help, because I don’t have a good experience of my country. There, when women go to hospital in pain, they are treated very badly (Maracaibo).*

Satisfaction with the treatment received by the team in both places was demonstrated, and was directly related to the perception of being cared for. Care was described as the presence, attention, listening and action of the professional, mostly in the face of moments of vulnerability and pain. Nursing care, in terms of clinical assessment of the woman and baby, practical aspects such as umbilical stump care, hygiene, breastfeeding and feeling seen by the caring gaze, was cited positively in the childbirth experience in Brazil:

*I did my care with the nurses, and everything was great, great. They explained everything to me: how to breastfeed my baby, how to clean the navel, how to bathe him, his first bath. (...) my other son is one year and eight months old, he was born in Peru by C-section too, because he was sitting up. I liked it better here. The attention, the delivery, the stay, the cleanliness, it was very different. Today I feel happy (laughs), happy because they listened to me (Valencia)*

*They change the towel, hand it over, help me, take care of everything. If it hadn’t been for them, I think I’d be worse off. (...) I was having such a bad time, someone ran out to call the doctors. They got me up, took me back to the room, gave me medicine. Everything, everything, everything was just right. If it hadn’t been for them, I would have died (Delmas)*

Care, as well as providing momentary relief, made it possible to participate in the decision-making process for the birth. One employee used the word humanization when describing her care and related this concept to sympathy and respect in care. In the scenarios in which the employee was the protagonist, the reports were more positive:

*And one thing I really wanted was to take a photo with my daughter next to me. They (the staff in the room) help me. My cell phone was running out and one of them lent me his phone to take a picture. What a precious thing! They might have thought it was a waste of time, but they helped me a lot, a lot (Delmas).*

Regarding perceived barriers, there were negative interventions, including recurrent vaginal touching:

*The pain was unbearable and I wasn’t dilating. They had already done the touch twice and a brown liquid was coming back (...) I didn’t want to endure another touch; it was too painful (Valencia).*

Another barrier was the difficulty in communicating, reported as not understanding medical terms or ability in Brazilian Portuguese and the feeling of not being heard and understood, a barrier that was overcome as the care team adapted their speech. Although some collaborators communicated in Portuguese, the field notebook recorded their preference for communicating in their mother tongue:

*(...) the doctors here in my room, they explain well, they speak Portuguese slowly so that I understand well, because I’m a foreigner and they ask if I’ve understood (Tunis).*

## DISCUSSION

The main immigrant groups in Brazil are Haitians and Venezuelans. At Brazilian border entry points, women of Argentinian, American, French and Portuguese nationality were the most common. Although in smaller numbers, Paraguayan, Chilean, Uruguayan, Bolivian and Peruvian women have also been present, and towards the end of the last decade, the entry of Haitian and Venezuelan women are the most remarkable^(5)^. Immigrant women are a vulnerable group regarding health care^(3)^. However, these women have been able to access the Brazilian health system in recent years, especially maternal care services related to pregnancy, childbirth and the puerperium, medium-complexity services and tests in the territory^(2,4)^.

Immigrant women’s expectations around labor and birth are shaped by their experiences in their country of origin. These women have a diverse and multicultural lens on traditional childbirth and what they prefer^(9)^. On the international scene, there are reports of a preference for vaginal birth among immigrant women^(9-11)^. Regarding the childbirth experience of Japanese women in Bali, it was pointed out that most of them also expected to experience normal childbirth and that this route is normalized in their culture of origin, however some participants had to deal with the frustration of having a surgical delivery. What helped them overcome this frustration was the certainty that the baby was fine and that the team recommended surgery because it was necessary^(11)^.

In the Brazilian scenario, Syrian immigrant women living in São Paulo reported their desire for an elective C-section and their surprise at not being able to get one easily in the Brazilian system and this impossibility of choosing the route of delivery beforehand was impactful, as they were used to the culture of their country of origin, with Christian women in particular preferring a C-section to avoid pain^(4)^.

There are records of a higher occurrence of caesarean sections among immigrants, with the country of origin possibly being associated with a greater indication for this surgery^(10)^, including emergency caesarean sections^(12)^. In multiparous immigrant women, this route of birth is indicated because they have had previous C-sections^(13)^.

With regard to sensations and feelings, pain is a topic usually presented in studies on labor and birth. The event of pain is constructed as an interweaving of the threads of the body, language and culture. To understand pain would be tantamount to read what it conveys; it would be like appreciating the emergence of a figure in the weaving of embroidery^(1)^.

When investigating the cultural values linked to pain in childbirth and how these values influence this experience, just over half (56%) of the participants had an accurate answer as to why there is pain during labor, relating pain to the first and second stages of childbirth. The authors pointed out that six participants answered this question by linking ideological and religious aspects to pain in childbirth, such as a divine curse, karma and the like. They also found that 43% of the participants described pain using words of violence, with some relating the pain of childbirth to feelings of fear and loneliness^(14)^.

Contributing to this perception, Muslim puerperal women in São Paulo reported that they and their community preferred normal childbirth; pain is Allah’s permission, a divine predestination for that woman; to deny pain would be to deny what builds their being^(4)^. On the other hand, there have been reports of immigrant women who have had caesarean sections, but who could potentially have endured the pain of labor if they had received support and encouragement^(11)^. International studies differ in terms of immigrant women’s access to pharmacological and non-pharmacological analgesia^(10,15,16)^, but our study was limited to not delving into the perception of the collaborators about the use of analgesia.

Similar to our study, others have pointed out that fear among immigrant women is more often related to insecurity and lack of care support. The unfamiliar and different health system and care practices, the difficulty of registering and accessing information, language barriers^(11,16)^, loneliness and fear of mistreatment^(16)^, and care that is not centered on the woman are all factors that precede childbirth, but which feed the feeling of dissatisfaction^(11,16,17)^.

Immigrant women in childbirth are afraid of being treated differently by the staff. Immigrant women are often unaware of whether childbirth care will be similar to that in their country or whether their customs will be respected by the team, and the feeling of vulnerability and mistrust can be aggravated by the difficulty of communication^(16)^. Fear and frustration on the part of immigrant women when faced with different obstetric systems, whether they are more interventionist or more based on scientific evidence, have been pointed out by other studies^(11,18,19)^.

Poor, inhumane obstetric care has a persistent impact on women’s lives, causing many to relive and recount their previous childbirth experience in the present, reinforcing the idea that the childbirth experience is not static, but something organic, active and transformative; its meaning has the capacity to echo down to future generations^(20)^. The memories presented by the collaborators in this study describe a scenario of obstetric violence, with disrespect and indifference prevailing. The appropriation of women’s bodies occurs through abusive interventions, leading them to lose their autonomy and leading role, especially during childbirth^(2)^.

Despite the barriers reported in the childbirth experience, positive satisfaction as a superficial response to childbirth care is common in almost all scenarios. Immigrant women also appear to be more satisfied than native women with maternal health services^(21,22)^. At first, these women tend to report satisfaction with the perinatal care they received, but when the experiences are evaluated categorically, immigrant women complain about key aspects of care^(21)^.

In this respect, immigrant women from low socio-economic backgrounds in Germany had negative opinions about communication and dignity when they reflected in depth on their experience. In addition to impaired communication, the feeling of disrespect from professionals was aggravated by non-verbal interaction and derogatory comments about the community or nationality to which the woman belonged. Personal complaints such as delays in the process, the presence of foul-smelling fluids and fatigue on the part of the professional during the process also interfered negatively with the women’s sense of dignity, respect and empowerment^(21)^.

It is therefore necessary to discuss the care provided by the multi-professional team and the sense of care perceived by immigrant women. This differentiation is necessary because, among the mishaps in the communication process described above, the care provided is not always the care perceived and both have an impact on the woman’s perception of her childbirth process^(21,23)^.

In this study, the words used by the collaborators to describe perceived care were: “being close”, “concerned”, “calming”, “support” and “holding hands”. Similarly, Syrian women in Turkey also described moments of care with the references “they calmed me down”, “they cared”^(18)^. Immigrant women want to be cared for by professionals who provide security, encouragement, who are good listeners and who communicate information well. Above all, the professionals must have a respectful and welcoming attitude. Feeling safe and taken seriously is a fundamental part of caring for immigrant women due to their situation of vulnerability and fragility in relation to care. In general, these women have not experienced care in their recent life history. Care during childbirth is a bridge to a new perspective on life^(16)^.

Care is essential in nursing practice, i.e., it is essential to talk about nursing care to the immigrant women, as it is linked to a more positive and autonomous childbirth experience. Although they mentioned the professional team as a whole, it is relevant to present the work of obstetric nursing in this field, given its implications for professional practice. The inclusion of the obstetric nurse in this care setting has strengthened good practices in obstetrics, recommended by the World Health Organization, and it is up to the professionals who provide care to value the physiological aspects involved in pregnancy and parturition, avoiding unnecessary interventions and offering qualified and safe care^(24)^.

In this sense, barriers such as communication difficulties directly interfere with care, generating dissatisfaction when there is a lack of information about the pregnancy-puerperium cycle and when it is not in accessible language and format. Adequate care involves a relationship of trust between women and health professionals, as it prepares women and helps them to feel confident and prepared for childbirth; this care is expressed through an encouraging and reassuring presence, professionals being attentive to listening to women, providing appropriate information, being culturally sensitive and having a strong knowledge of immigrant beliefs and traditions^(16)^. What is known as responsive communication with care and empowerment is developed, which includes the non-verbal communication of the team, who are welcoming, patient and attentive when they notice that the woman has any doubts or feels any kind of fear during labor. This attitude on the part of the team promotes respect, safety, a sense of understanding and appreciation of the individual^(19)^.

The use of medical jargon and the inconsistency of the information received by different professionals has been shown to hinder the communication process during childbirth for immigrant women. Faced with the intensity of the internal childbirth process and the difficulty of external communication, immigrant women reported the need to increase their voice volume with the team^(21)^.

Communication should be a channel that facilitates care between nurses and parturients. It is the path of exchange between the subjects, allowing a relationship whose ultimate purpose is the woman’s need, which often means being present, paying attention, speaking and gesturing, and small gestures such as hand contact or the use of a warm compress. In this sense, effective communication promotes autonomy and independence in the birthing process^(25)^. In Brazil, some services that care for immigrant women provide opportunities for their professionals to learn the language of that community or offer or give lectures on the culture of that group. However, not all professionals do this, and the constant need to communicate with a non-Portuguese-speaking group, either in another language or by gestures, seems to irritate some professionals^(26)^.

Information in the mother tongue is also a safety enhancer. Countries that have invested in information appropriate to the language needs of different immigrant groups provide printed materials in the language of their users, translation channels via telephone or translators, and a greater focus on clarifying doubts during consultations. In addition, parturients have sought out internet channels to translate terms, access materials in their own language and support through multicultural communities^(11,16,19)^.

The municipality of Curitiba provides the *Carteira da Gestante* (Pregnancy Passport) with information about prenatal care, pregnancy, childbirth and breastfeeding in the four most widely spoken languages in the city: Portuguese, Spanish, English and Creole. Encouraging well-informed pregnant women is in line with the municipality’s aim of providing humanized care to all women, including immigrants. However, information in the same languages is not found in hospital care^(27)^.

It is therefore understood that immigrant women’s experience of labor and birth begins well before the moment of delivery. Their bodies pulsate with their history, their culture and their country of origin. Therefore, caring for these women, respecting their customs and building expectations must begin during prenatal care. The relationship with the care team must be based on trust and understanding on both sides. It is possible to provide humanized and individualized care to immigrant women. Intelligible, calm and respectful communication is essential throughout the process, because through information immigrant women gain security and autonomy over their childbirth process.

The study’s limitations include the fact that it was limited to just one Brazilian location, but it still provides important contact with the real story revealed by immigrant women about their childbirth and postpartum experience, providing valuable and useful information for a care practice that takes into account the cultural aspects involved in the labor and birth process of immigrant women, an increasingly growing population in the country. The occurrence of the COVID-19 pandemic in the midst of data collection restricted the number of participants and free access to maternity hospitals. However, there was no direct mention of the pandemic or the safety protocols of that period by the collaborators. Thus, this research was limited to not investigating in depth the impact of the pandemic on maternal care for immigrant women, which is recommended as a topic for future studies.

## CONCLUSION

The study allowed to know the labor and birth process of the immigrant women who collaborated in the research, as well as the barriers in this process and their perceptions of the care they received in the services, showing how nursing care takes place. Understanding labor, birth, pain and the symbolism involved makes it easier for nurses to look at immigrant women with patience and complexity. Being together, communication, respect and dignity are values that should be defended by the nurse when caring for an immigrant woman giving birth. In this context, as a strategy, maternity wards can provide visual resources in the main foreign languages on relaxation techniques and non-pharmacological methods for pain relief.

This study showed that it is necessary to improve the training of nurses in transcultural care, from their initial academic training to their specialization and continuing health education. In order to improve labor and birth care for immigrant women, more research is needed on perinatal indicators, the birth experience of other immigrant groups and their birth culture.

Assistance during the labor and birth process can be experienced through quality nursing care, with good communication, cultural sensitivity and welcoming of the various needs. Immigrant women can be the protagonists of their lives, of the choices they make about their bodies, and of the culture they bring with them, thus increasing their security and strength.
